# Loss of SUSD2 expression correlates with poor prognosis in patients with surgically resected lung adenocarcinoma

**DOI:** 10.7150/jca.39319

**Published:** 2020-01-14

**Authors:** Wei Guo, Fei Shao, Sijin Sun, Peng Song, Lei Guo, Xuemin Xue, Guochao Zhang, Hao Zhang, Yibo Gao, Bin Qiu, Fengwei Tan, Shugeng Gao, Jie He

**Affiliations:** 1 Department of Thoracic Surgery, National Cancer Center/National Clinical Research Center for Cancer/Cancer Hospital, Chinese Academy of Medical Sciences and Peking Union Medical College, Beijing 100021, The People's Republic of China; 2 Department of Pathology, National Cancer Center/National Clinical Research Center for Cancer/Cancer Hospital, Chinese Academy of Medical Sciences and Peking Union Medical College, Beijing 100021, The People's Republic of China; 3 The Affiliated Hospital of Qingdao University and Qingdao Cancer Institute, Qingdao, Shandong 266071,The People's Republic of China

**Keywords:** Lung adenocarcinoma, Sushi Domain Containing 2, Prognosis, Immunohistochemistry, Biomarker

## Abstract

There is limited evidence regarding the relationship between the expression of Sushi Domain Containing 2 (SUSD2) and prognosis of patients with surgically resected lung adenocarcinoma (LUAD). This retrospective study aimed to investigate the clinical significance of SUSD2 expression in LUAD. To assess SUSD2 expression in LUAD, we conducted both integrated bioinformatic analysis based on the TCGA database and also immunohistochemistry study using a tissue microarray encompassing 578 LUAD cases from our hospital. Reduced SUSD2 expression was associated with gender, smoking history, higher pathological grade, lymph node metastasis, larger tumor length, advanced TNM stage. LUAD patients with SUSD2-positive tumors showed significantly better overall survival (OS) than those with SUSD2-negative tumors (P = 0.000). When patients were stratified into those with stage I (218, 37.7%), II (152, 26.3%) and III (208, 36.0%) disease, and those without (254, 43.9%) and with (324, 56.1%) lymph node metastasis, the prognostic effect was almost consistent. The OS of patients with positive SUSD2 expression was significantly better in patients with stage I (P = 0.000), III (P = 0.000), without (P = 0.000) and with (P = 0.001) lymph node metastasis. Multivariate analysis showed that loss of SUSD2 predicted a shorter survival time and was an independent prognostic factor for LUAD patients. Our study indicated that SUSD2 may serve as a new prognostic and potential therapeutic target in LUAD.

## Introduction

Lung cancer remains the most commonly diagnosed cancer and the leading cause of cancer death worldwide 1. Among all lung cancer patients, lung adenocarcinoma (LUAD) accounts for the more than 50% of cases and its incidence is still increasing 2, 3. Although in recent years we have made significant breakthroughs in surgery, chemotherapy, molecular targeted therapy, immunotherapy, and radiotherapy, the long-term outcomes of LUAD patients remain poor 4. Currently, the prognosis of LUAD patients is mainly predicted by the pathology-based TNM stage and pathologic classification, which does not provide sufficiently detailed information to delineate definitive clinical outcomes in patients with LUAD. Therefore, there remains an unmet clinical need for biological markers that can more precisely stratify patients about long-term prognosis.

Sushi domain containing 2 (SUSD2) is located on chromosome 22 and encodes an 822-amino acid type I transmembrane protein, consisting by somatomedin B, AMOP, von Willebrand factor type D, and Sushi domains 5. In 2007, two studies by Sugahara *et al.* demonstrated a potential tumor suppressive function of the mouse homolog SUSD2 6, 7. In 2013, Watson *et al.* reported that SUSD2 promoted many aspects of breast cancer tumorigenesis, including tumor immune evasion, angiogenesis, and metastasis 5. Recently, SUSD2 has been studied in the context of different kinds of solid tumors 8-15, including lung cancer 16, 17. Some of the aforementioned studies indicated that SUSD2 played a tumor suppressive role in tumorigenesis. High expression of SUSD2 may inhibit tumor cell proliferation, migration, and invasion in high-grade serous ovarian cancer, hepatocellular carcinoma (HCC), lung cancer and renal cell carcinoma 8, 13, 16. However, there were also some studies reported that SUSD2 played a tumor oncogene role in tumorigenesis. Umeda *et al.* reported downregulation of SUSD2 might reduce the proliferation, migration, and invasiveness of gastric cancer cells. Moreover, in gastric cancer patients with high SUSD2 expression, the incidence of hepatic recurrence was much greater 12. Xu Y reported high expression of SUSD2 in ovarian cancer cells contributed to epithelial-mesenchymal transition (EMT) and the metastatic capacity of malignant cells 9. Elizabeth *et al.* demonstrated that SUSD2-expressing breast cancer cells potentiated angiogenesis indirectly by the recruitment of macrophages into the tumor by secretion of by secreting factors that directly stimulated endothelial vessel formation 15. Taken together, SUSD2 had complex functions and SUSD2 likely regulated specific signal transduction processes determined by the cell type and the state of cell differentiation or pathology.

As for lung cancer, related research is still limited. Cheng Y *et al.* reported that SUSD2 was frequently decreased in lung cancer tissues compared with the corresponding levels in normal adjacent tissues. The restoration of SUSD2 expression inhibited the proliferation and clonogenicity of lung cancer cells 16. Cai C *et al.* reported that reduced SUSD2 protein levels in cancer tissues were positively correlated with poor histological grade, advanced clinical stage, higher pathological T stage and positive regional lymph node metastasis in NSCLC 17. The two published work suggested that overexpression of SUSD2 may be an important tumor suppressor in tumorigenesis of lung cancer. However, to the best of our knowledge, little is known about the effect of SUSD2 expression on the survival of LUAD patients.

To elucidate the effect of SUSD2 expression on the survival of LUAD patients, we first performed an integrated bioinformatics analysis based on the Cancer Genome Atlas (TCGA) database. We compared the expression levels of SUSD2 in tumor tissues of different pathological stages and also non-tumor tissues. We examined the correlation between SUSD2 expression and overall survival (OS) of LUAD patients. Then, we used immunohistochemical (IHC) analysis to explore the prognostic value of SUSD2 in our institutional large-scale LUAD cohort. We examined the correlation between SUSD2 expression level with patients' clinicopathological variables and OS. These results indicated that reduced expression of SUSD2 was correlated to progressive features and SUSD2 was one independent prognostic factors of OS for patients with surgically resected LUAD.

## Materials and Methods

### Patient Samples

The specimens in this study, tumor tissues with corresponding noncancerous tissues were collected from the 578 patients with LUAD who underwent R0 resection between June 2006 and June 2014.

**Figure 1:**
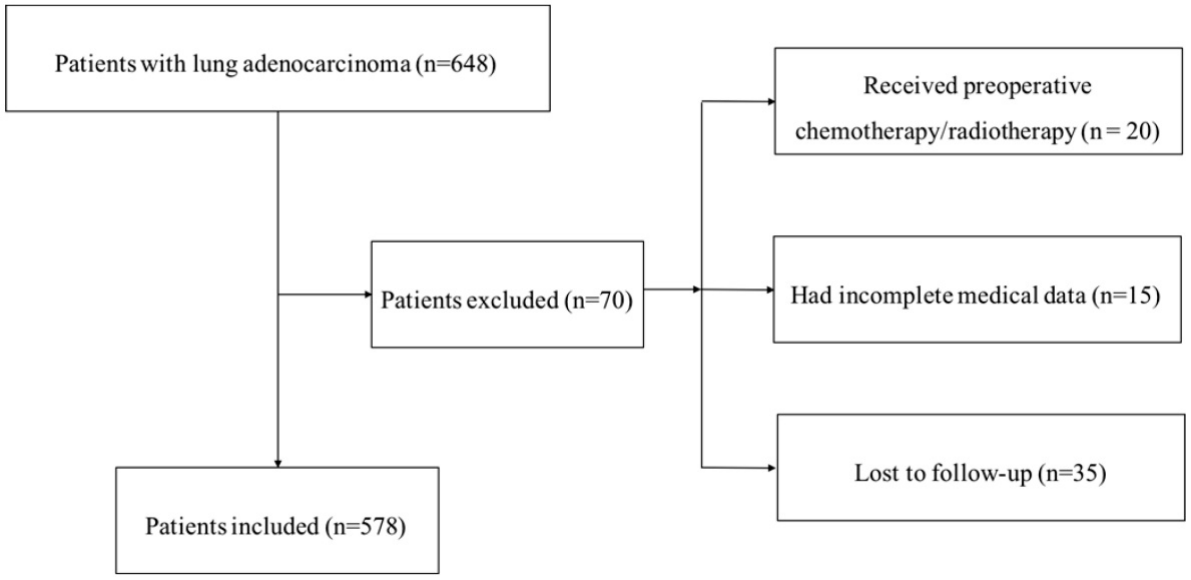
The flowchart of the enrollment process

Our institutional database of medical records of 648 consecutive patients with surgically resected LUAD were retrospectively reviewed. The inclusion criteria were as follows: (1) radical surgery with R0 resection; (2) histologically confirmed LUAD. The exc lusion criteria were as follows: (1) if the patients received preoperative chemotherapy and (or) radiotherapy; (2) if the patients lacked detailed clinical information; (3) if the patients lost to regular follow-up. Seventy patients were excluded from this study. The whole enrollment process was clearly shown in Figure 1. Among these patients, 20 patients received preoperative chemotherapy and (or) radiotherapy; 15 patients had incomplete medical data; and 35 patients were lost to follow-up. Finally, a total of 578 patients were enrolled in the present study.

All patients provided informed consent before surgery. The clinicopathological data, including age, gender, tumor location, tumor differentiation status, T stage, lymph node metastasis and TNM stage of LUAD patients were recorded. All of the specimens were pathologically confirmed by two pathologists. The pathological classification of the primary tumor and the degree of lymph node metastasis were confirmed according to the 8^th^ TNM stage 18.

The study was conducted following the Declaration of Helsinki. The Clinical Research Ethics Committee of National Cancer Center/Cancer Hospital, CAMS approved this study. Patients were followed up in the outpatient department regularly (every 3-6 months) for the first two years after surgery and then annually. The follow-up included documentation of the patients' medical history, physical examinations, and chest computed tomography. The last follow-up was on March 4, 2019.

### SUSD2 Expression analyses in TCGA Database

To investigate the expression and prognostic value of SUSD2 as well as correlation between SUSD2 expression and key genes mutation in LUAD, we extracted SUSD2 expression, clinical information and key genes SNP information from TCGA GDC data portal (https://portal.gdc.cancer.gov/). A total of 535 LUAD patients were included in this analysis. Box Plots were used to compare the expression level of SUSD2 in LUAD tumor tissues and normal tissues as well as the expression of SUSD2 in tumor tissues of patients with different pathological stages, Point-Line plot was applied to illustrate the expression level of SUSD2 between tumor tissues and pairing normal tissue. The Kaplan-Meier curves were used to analyzed patients' survival according to the expression level of SUSD2.

### GEPIA analysis of SUSD2 expression

GEPIA (http://gepia.cancer-pku.cn/index.html) is a newly developed online software, which is commonly used for analyzing certain genes expression differences between cancer and normal tissues in various tumor types. In the present study, GEPIA was used to analyze the expression level of SUSD2 between LUAD tumor tissues and normal lung tissues.

### SUSD2 Expression and survival analyses in KM-PLOT lung cancer Database

The Kaplan-Meier plotter (http://kmplot.com/analysis/) is an online tool applied to assess the effect of 54,675 genes on survival using 10,461 cancer samples. The Kaplan- Meier plotter mRNA lung cancer database was applied to evaluate the prognostic values of SUSD2 in patients with LUAD. In our study, LUAD patients were screened out based on the TNM stage and lymph node metastasis status. Patients with LUAD were divided into two groups according to the median values of mRNA expression.

### Immunohistochemistry (IHC) of SUSD2

The tissue microarrays (TMAs) were constructed by tissue blocks of 578 cases from biobank of our hospital. A serial of 4-μm-thick sections were cut and transferred to adhesive slides according to manufacturer's instructions. Briefly, TMAs were deparaffinized, rehydrated, treated with 2N HCl for 15 min, and treated with 100 mM Tris-HCl (pH 8.5) for 10 min. Subsequently, the sections were blocked with 3% H_2_O_2_ for 30 mins and goat serum at room temperature for 30 mins. After blocking, the sections were incubated with rabbit anti-SUSD2 polyclonal antibody (1:2000, HPA004117, Sigma-Aldrich, St Louis, MO, USA) at 4°C overnight and then incubated with polyclonal peroxidase-conjugated anti-rabbit IgG (Zhongshanjinqiao, Beijing, China) at room temperature for 20 min according to the manufacturer's instructions.

### Evaluation of immunostaining

**Figure 2:**
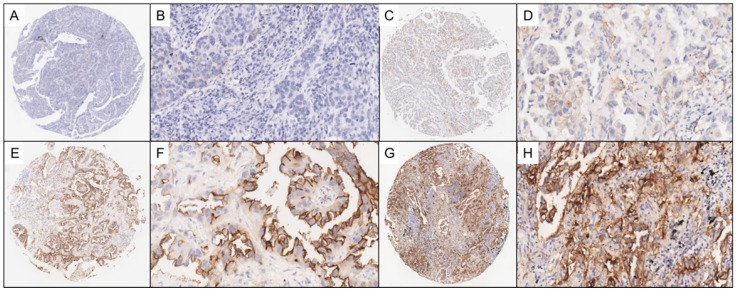
Representative photomicrographs of lung adenocarcinoma (LUAD) TMA sections. Figure 2A-2B show representative photomicrographs stained with SUSD2 which score = 0. Figure 2C-2D show representative photomicrographs stained with SUSD2 which score = 1. Figure 2E-2F show representative photomicrographs stained with SUSD2 which score = 2. Figure 2G-2H show representative photomicrographs stained with SUSD2 which score = 3.

Two experienced pathologists who were blinded to the clinical data independently reviewed IHC staining of the LUAD TMAs and scored each tissue sample based on the percentage of tumor cells stained for SUSD2, range from 0 to 3. The scoring distribution is defined as the following: a score of 0, no SUSD2 staining; 1, <10% positive SUSD2 staining; 2, 10-50% positive SUSD2 staining; 3, >50% positive SUSD2 staining. In this study, tissues with scores ranging from 0 to 1 were grouped and classified as having negative levels of SUSD2, whereas tissues with scores ranging from 2 to 3 were grouped and classified as having positive levels of SUSD2. In Figure 2, we illustrated the representing IHC staining of SUSD2 in the specimens.

### Statistical Analysis

Statistical analyses were performed by SPSS 23.0 (IBM Corporation, New York, USA). Cross tabulations of clinical data and marker expressions were analyzed using the Chi-square test or Fisher's exact test. We used the Kaplan-Meier method to analyze overall survival (OS). Risk factors for the prognosis of LUAD patients were calculated by univariate Cox regression, and those with P values up to 0.05 were included in a multivariate Cox regression to identify independent prognostic variables. A P value below 0.05 was considered statistically significant.

## Results

### Aberrant SUSD2 down-regulation in LUAD cancer

We analyzed the expression profile of SUSD2 in different types of human cancers using the Oncomine database. The results indicated that SUSD2 expression was lower in some types of tumors including Lung cancer, colorectal cancer, esophageal cancer, gastric cancer, and sarcoma comparing to their matched normal tissues (Figure 2A). Oncomine analysis of cancer vs normal samples revealed that SUSD2 expressed statistically significantly higher in LUAD comparing to normal tissues (Table 1).

However, in brain and CNS cancer, breast cancer, liver cancer, SUSD2 expression was lower. Moreover, GEPIA analysis was performed to investigate the expression of SUSD2 in various human tumors, the results also showed that SUSD2 expression level was lower in some types of tumors including LUAD (Figure 2B, Figure 2C).

**Figure 3:**
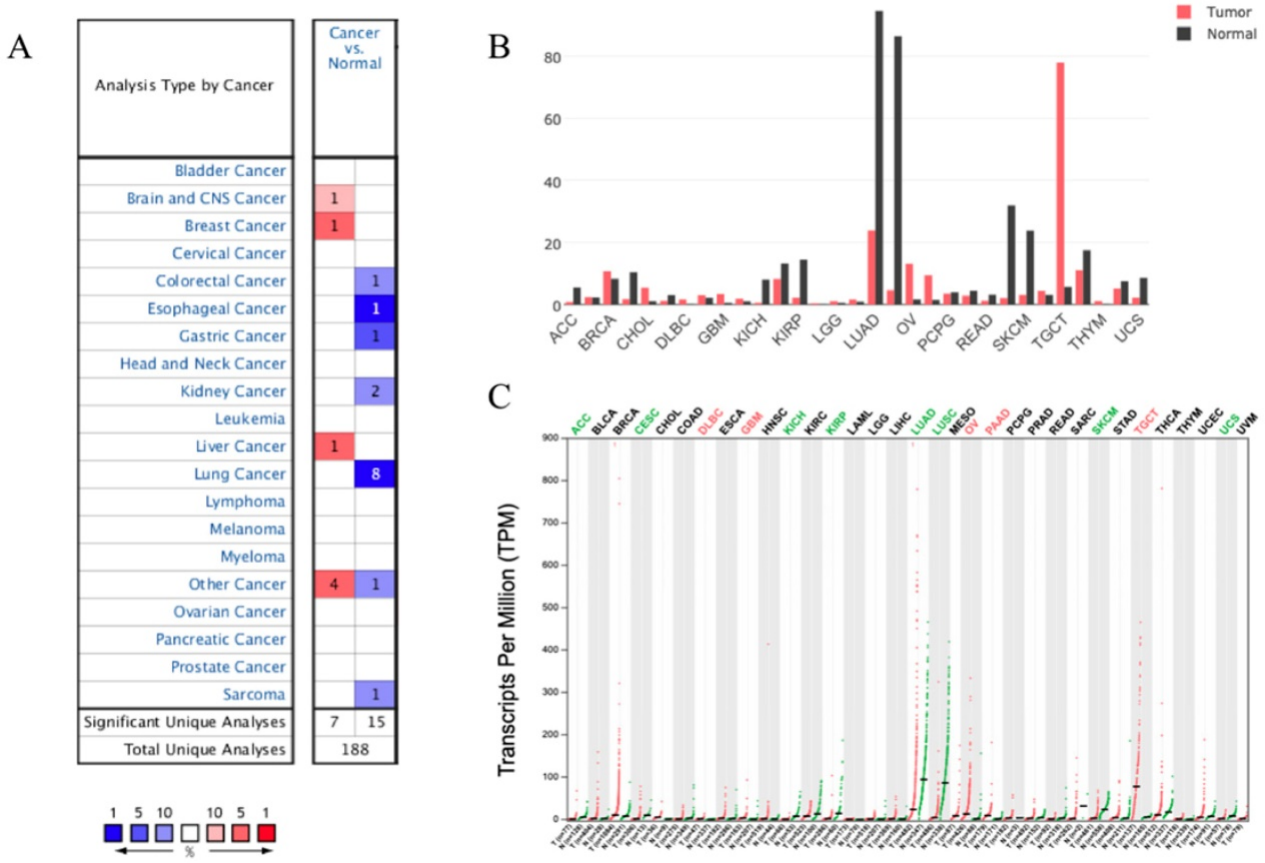
Expression of SUSD2 in different types of human cancers (A) Expression of SUSD2 in different types of human cancers in Oncomine database; (B) Expression of SUSD2 in different types of human cancers by GEPIA analysis (Dot plot); (C) Expression of SUSD2 in different types of human cancers by GEPIA analysis (Bar plot).

We then analyzed the SUSD2 expression level of the LUAD cases from the TCGA database and there was a significant difference between the LUAD tumor tissues and normal tissues (Figure 3A, Figure 3B). Meanwhile, the average expression level of SUSD2 turned out to have a gradually decreasing trend as the development of the TNM pathological stage in stage I-III (Figure 3C). The OS of ESCC patients according to SUSD2 expression are shown in Figure 3D. High SUSD2 expression is significantly associated with better OS (p = 0.0042).

### Correlations between SUSD2 expression and clinicopathological parameters of LUAD patients

In Table 2, we summarized the correlations between SUSD2 expression and clinicopathological parameters of LUAD patients. The median age of the LUAD patients at diagnosis was 66 years, ranging from 25 to 86. Three hundred and twenty-five patients (56.2%) were male, and 253 patients (43.8%) were female. The median follow-up time was 50.6 months (0.85-124.36months), and 328 patients (56.7%) died during follow-up. The SUSD2 expression had a significant correlation with the clinicopathological parameters such as gender, smoking history, differentiation grade, tumor length, T stage, N stage and TNM stage (all P < 0.05).

### Survival analysis of SUSD2 expression for LUAD patients

The OS of LUAD patients according to SUSD2 expression was shown in Figure 3A. The result demonstrated that the positive SUSD2 group had a significantly better five-year OS than the negative SUSD2 group (p < 0.001).

Next, the univariate analysis and the multivariate analysis were used to investigate the risk factors for OS of the LUAD patients. In the univariate analysis, the results showed that age, gender, smoking history, tumor length, T stage, lymph node metastasis, TNM stage and SUSD2 expression were associated with the OS of LUAD patients (all P < 0.05) (Table 3). Then, we used the multivariate analysis to investigate the independent risk factors. Age, smoking history, tumor length, lymph node metastasis, TNM stage and SUSD2 expression were independent prognostic factors of OS for LUAD patients (all P < 0.05) (Table 3).

**Figure 5:**
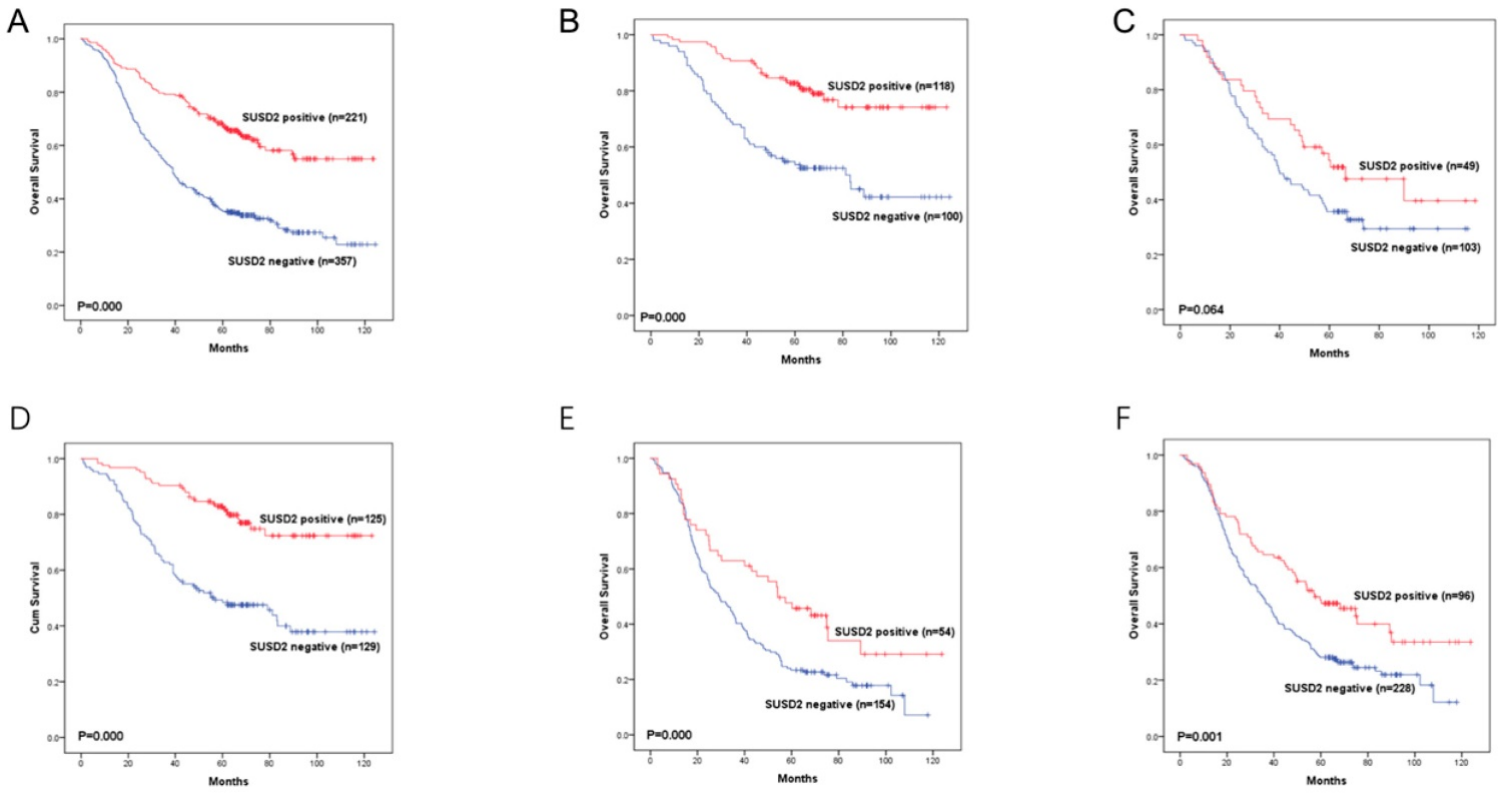
Kaplan-Meier curves showing survival of the 578 patients with LUAD according to SUSD2 expression.

Moreover, we divided the 578 LUAD patients into different groups according to their lymph node status or TNM stage. In LUAD patients with TNM stage I or stage III, high SUSD2 expression was associated with better OS (P = 0.000, 0.006, respectively) (Figure 5B, 5D). However, in LUAD patients with TNM stage II, there was no statistically significant difference between these two groups (P = 0.064) (Figure 5C). In patients without lymph node metastasis, high SUSD2 expression was associated with improved OS (P = 0.000) (Figure 5E). In LUAD patients with lymph node metastasis, high SUSD2 expression was also associated with better OS (P = 0.001) (Figure 5F).

**Figure 6:**
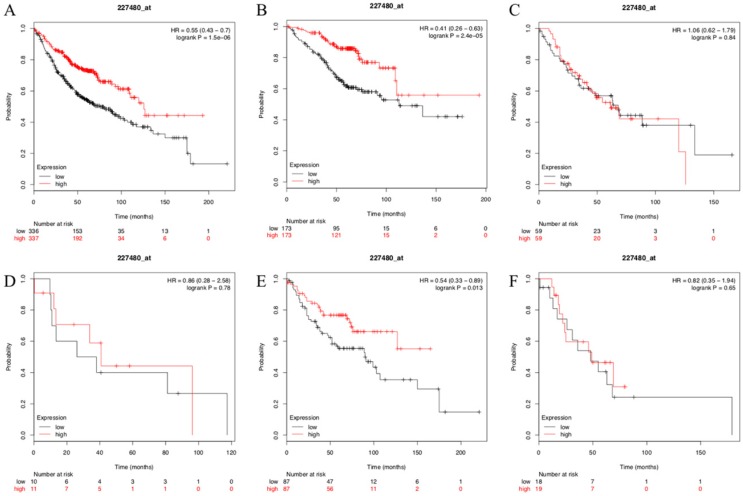
Kaplan-Meier curves showing survival of the 673 patients with LUAD in KM-PLOT database according to SUSD2 expression.

To test and verify our results, the Kaplan- Meier plotter mRNA lung cancer database was used to evaluate the prognostic values of SUSD2 in patients with LUAD. A total of 673 LUAD patients were available for the analysis. We found that high expression of SUSD2 had a significantly better OS and SUSD2 expression was independent prognostic factors of OS for LUAD patients (Figure 6A). We also divided the 673 LUAD patients into different groups according to their lymph node status or TNM stage. In LUAD patients with TNM stage I, high SUSD2 expression was associated with better OS (P < 0.001) (Figure 6B). However, in LUAD patients with TNM stage II and III, there was no statistically significant difference (Figure 6C, Figure 6D). In patients without lymph node metastasis, high SUSD2 expression was associated with improved OS (P = 0.013) (Figure 6E). However, In LUAD patients with N1 lymph node metastasis, high SUSD2 expression was not associated with better OS (P = 0.65) (Figure 6F).

## Discussion

SUSD2 is type I membrane protein containing domains inherent to adhesion molecules 6, 7. In previous studies, we found that SUSD2 is a gene with unclear functions. Several studies have reported the role of SUSD2 in cancer and SUSD2 seemed to sever as a valuable factor for tumorigenesis in different types of cancer 5, 8-11, 14-17.

In many types of cancer, SUSD2 are considered to act as a tumor suppressor 8, 11, 16, 17. Cheng Y *et al.* reported that the expression of SUSD2 was frequently decreased in lung cancer tissues compared with the corresponding normal tissues. overexpression of SUSD2 could inhibit the proliferation and clonogenicity of RCC and lung cancer cells, whereas knockdown of SUSD2 could promote lung cancer cell growth 16. Cai C *et al.* also reported that the expression of SUSD2 was also significantly decreased in NSCLC tissues compared with those of adjacent normal tissues. The reduced SUSD2 expression level in lung cancer tissues was positively correlated with poor histological grade, advanced clinical stage and positive regional lymph node metastasis 17. In HCC, Liu XR *et al.* showed that decreased expression of SUSD2 was also observed in the majority of HCC tissues, compared with paired normal liver tissues. Knockdown of SUSD2 could promote HCC cell proliferation, invasion and migration, reduced the cell apoptosis. Moreover, the reduced SUSD2 expression level in HCC tissues was positively correlated with high pathological grade, advanced clinical stage, lymph node metastasis and distant metastasis 8. In high-grade serous ovarian cancer, Sheets JN demonstrated that SUSD2 impedes migration, EMT and mesothelial clearance of cancer cells, consistent with prolonged survival of patients with SUSD2-positive tumors 11. However, in many types of cancer, SUSD2 can also act as a tumor-promoting gene 10, 12, 14, 15. Different from Sheets JN's results, Xu Y *et al.* reported that overexpression of SUSD2 in ovarian cancer cells promoted EMT and the metastatic capacity of malignant cells. In contrast, silencing SUSD2 in aggressive ovarian cancer cells inhibited these processes both in vitro and in vivo 9. Moreover, Larger TW *et al.* reported that SUSD2 could inhibit platelet activation and binding to high-grade serous ovarian carcinoma cells thus inhibit platelet driven mechanisms of cancer cell progression, such as metastasis. High expression of SUSD2 correlated with longer survival in patients with high-grade serous ovarian carcinoma 10. In gastric cancer, Umeda S found that knockdown of SUSD2 could significantly reduce the proliferation, migration, and invasiveness of gastric cancer cells. High SUSD2 expression was significantly correlated with shorter survival in patients with gastric cancer 12. In endometrial cancer, Zhang S *et al.* reported that downregulation of SUSD2 causes cancer cell senescence and apoptosis 14. In general, the result on the role of SUSD2 in cancer remains controversial and it seems that SUSD2 can regulate different signal transduction processes determined by the cell types.

Currently, to the best of our knowledge, there is no paper for the prognostic value of SUSD2 in patients with surgically resected LUAD. Therefore, we conduct this present study to investigate the clinical significance of SUSD2 in our large-scale cohort. In this study, we showed that the expression of SUSD2 is downregulated at both the mRNA and protein levels in LUAD tumor tissues using bioinformatic analysis and IHC. To the best of our knowledge, we firstly verified that SUSD could be a potential prognostic biomarker in patients with surgically resected LUAD in both the TCGA cohort, KM-plot lung cancer mRNA database and ] our single institutional large-scale cohort. Firstly, we detected the SUSD2 mRNA level in the Oncomine database, GEPIA analysis and the TCGA cohort. We found SUSD2 mRNA level in the LUAD tumor tissues was significantly lower than that in adjacent normal tissues. Meanwhile, the average SUSD2 mRNA level turned out to have a gradually decreasing trend as the increasing of the TNM pathological stage in stage I-III. Then we tested the prognostic value of SUSD2 mRNA in TCGA cohort. High SUSD2 mRNA level was associated with better OS in LUAD patients. We then performed IHC staining in 578 cases of patients with surgically resected LUAD and we found that reduced expression was associated with the clinicopathological parameters such as gender, smoking history, higher pathological grade, regional lymph node metastasis, larger tumor length, advanced TNM stage and better OS. SUSD2 expression was an independent prognostic factor for OS in patients with surgically resected LUAD. Moreover, we used the Kaplan- Meier plotter mRNA lung cancer database to test and verify our results, the consistent results showed that high expression of SUSD2 had a significantly better OS and SUSD2 expression was independent prognostic factors of OS for LUAD patients.

In summary, we for the first time demonstrated that loss of SUSD2 predicted a shorter survival time and was an independent prognostic factor for LUAD patients. Our study indicated that SUSD2 may serve as a new prognostic and potential therapeutic target in LUAD. Whether SUSD2 functions as a tumor suppressor in LUAD and how are still worth studying in the future.

## Figures and Tables

**Figure 4:**
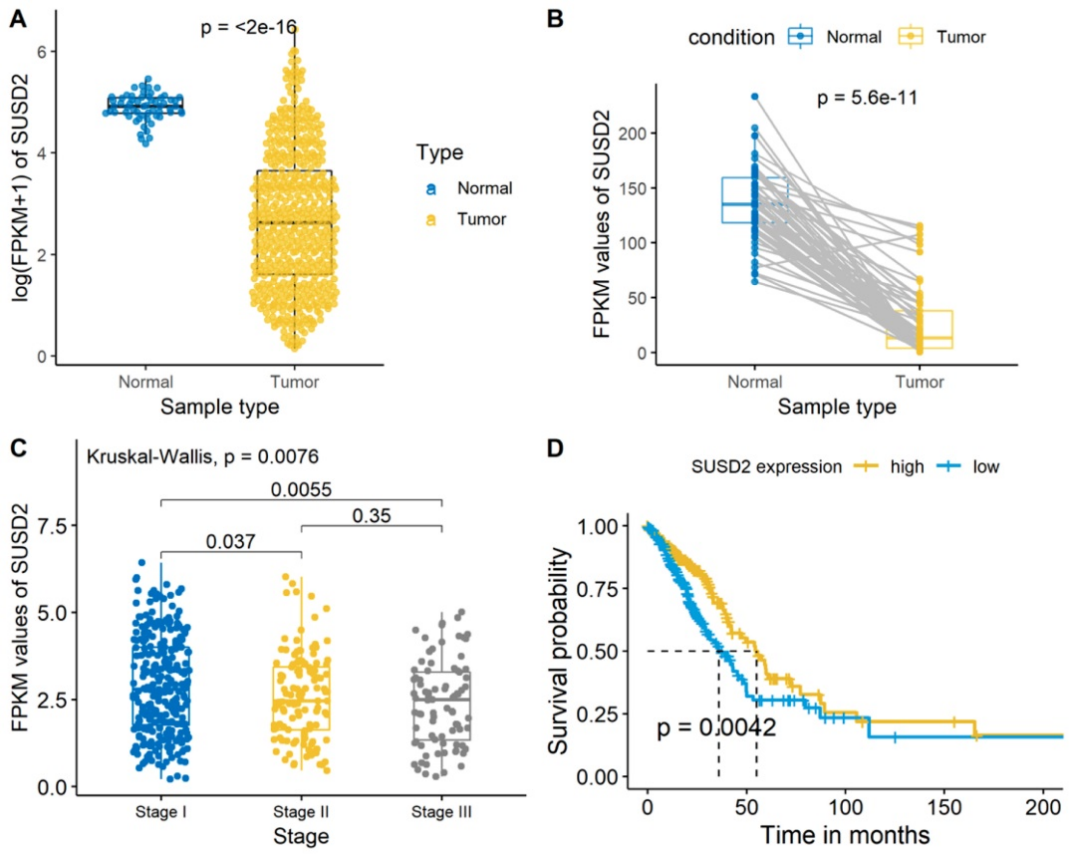
SUSD2 expression analysis and survival analysis of LUAD patients in TCGA database

**Table 1:** The significant changes of SUSD2 expression in transcription level between lung adenocarcinoma and normal tissues (ONCOMINE database)

References	P-value	Fold change	Rank (%)	Tumor	Normal
Hou	1.44E-15	-7.793	3	45	65
Selamat	2.65E-13	-9.103	7	58	58
Okayama	7.45E-8	-2.072	10	226	20
Garber	0.005	-3.713	11	40	5

**Table 2:** Correlations between SUSD2 expression and clinicopathological parameters of 578 patients with LUAD

Category	Cases (number, %)	SUSD2 expression		*P* value
	578 (100%)	Low (n=357)	High (n=221)	
Age (years)				0.864
≤60	291 (50.3)	181	110	
>60	287 (49.7)	176	111	
Gender				**0.000**
Male	325 (56.2)	227	98	
Female	253 (43.8)	130	123	
Smoking				**0.001**
Ever	303 (52.4)	167	136	
Never	275 (47.6)	190	85	
Tumor length (cm)				**0.000**
≤4	395 (68.3)	217	178	
>4	183 (31.7)	140	43	
Differentiation				**0.000**
Well	117 (20.2)	59	58	
Moderate	249 (43.1)	139	110	
Poor	212 (36.7)	159	53	
T stage				**0.000**
T1	210 (36.3)	95	115	
T2	263 (45.5)	176	87	
T3	63 (10.9)	53	10	
T4	42 (7.3)	33	9	
N stage				**0.000**
N0	254 (43.9)	729	125	
N1	156 (27.0)	108	48	
N2	161 (27.9)	114	47	
N3	7 (1.2)	6	1	
TNM stage				**0.000**
I	218 (37.7)	100	118	
II	152 (26.3)	103	49	
III	208 (36.0)	154	54	

**Table 3:** Univariate analysis and multivariate analysis of risk factors for prognosis of 578 LUAD patients

	Univariate analysis			Multivariate analysis		
	P value	HR	95%CI	P value	HR	95%CI
Age (≤60, >60years)	**0.002**	1.423	1.144-1.769	**0.000**	1.555	1.248-1.936
Gender (female, male)	**0.010**	1.339	1.073-1.670	0.506	1.125	0.794-1.595
Smoking (ever, never)	**0.000**	1.495	1.204-1.858	**0.048**	1.408	1.003-1.975
Tumor length (cm)						
≤4						
>4	**0.000**	2.058	1.651-2.566	**0.008**	1.414	1.094-1.829
Differentiation (well/moderate, poor)	0.112	1.190	0.954-1.485			
T stage (T1/T2, T3/T4)	**0.000**	2.060	1.602-2.650	0.809	1.040	0.759-1.425
lymph node metastasis (negative, positive)	**0.000**	2.394	1.891 -3.031	**0.005**	1.686	1.254-2.266
TNM stage (I/II, III)	**0.000**	2.259	1.818-2.808	**0.035**	1.375	1.022-1.849
SUSD2 expression (negative, positive)	**0.000**	0.409	0.319-0.525	**0.000**	0.524	0.403-0.683

^*^P less than 0.05 is significant.
